# Differential Bioaccumulation Patterns of α, β-Hexachlorobenzene and Dicofol in Adipose Tissue from the GraMo Cohort (Southern Spain)

**DOI:** 10.3390/ijerph19063344

**Published:** 2022-03-11

**Authors:** Inmaculada Salcedo-Bellido, Esperanza Amaya, Celia Pérez-Díaz, Anabel Soler, Fernando Vela-Soria, Pilar Requena, Rocío Barrios-Rodríguez, Ruth Echeverría, Francisco M. Pérez-Carrascosa, Raquel Quesada-Jiménez, Piedad Martín-Olmedo, Juan Pedro Arrebola

**Affiliations:** 1 Departamento de Medicina Preventiva y Salud Pública, Universidad de Granada, 18071 Granada, Spain; isalcedo@ugr.es (I.S.-B.); celiacazorla96@correo.ugr.es (C.P.-D.); anabel1993@correo.ugr.es (A.S.); prequena@ugr.es (P.R.); rbarrios@ugr.es (R.B.-R.); ruth.ecor@gmail.com (R.E.); fperezcar@gmail.com (F.M.P.-C.); 2 Consortium for Biomedical Research in Epidemiology and Public Health (CIBERESP), 28029 Madrid, Spain; 3 Instituto de Investigación Biosanitaria (ibs. GRANADA), 18014 Granada, Spain; eamayag@gmail.com (E.A.); fervs@ugr.es (F.V.-S.); raqueluna77@gmail.com (R.Q.-J.); piedad.martin.easp@juntadeandalucia.es (P.M.-O.); 4 Biomedical Research Center (CIBM), University of Granada, 18016 Granada, Spain; 5 Servicio de Oncología Radioterápica, Hospital Universitario Virgen de las Nieves, 18014 Granada, Spain; 6 Andalusian School of Public Health (EASP), 18011 Granada, Spain

**Keywords:** α-hexachlorocyclohexane, β-hexachlorocyclohexane, dicofol, human adipose tissue, exposure assessment, predictors

## Abstract

To identify bioaccumulation patterns of α-, β- hexachlorocyclohexane (HCH) and dicofol in relation to sociodemographic, dietary, and lifestyle factors, adipose tissue samples of 387 subjects from GraMo cohort in Southern Spain were analyzed. Potential predictors of these organochlorine pesticides (OCP) levels were collected by face-to-face interviews and assessed by multivariable linear and logistic regression. OCPs were detected in 84.2% (β-HCH), 21.7% (α-HCH), and 19.6% (dicofol) of the population. β-HCH levels were positively related to age, body mass index (BMI), mother’s occupation in agriculture during pregnancy, living in Poniente and Alpujarras, white fish, milk and water consumption, and negatively related to being male, living near to an agricultural area, working ≥10 years in agriculture, and beer consumption. Detectable α-HCH levels were positively related to age, BMI, milk consumption, mother’s occupation in agriculture during pregnancy, and negatively with residence in Poniente and Alpujarras, Granada city, and Granada Metropolitan Area. Residence near to an agricultural area, smoking habit, white fish and water consumption, and living in Poniente and Alpujarras, Granada city and Granada Metropolitan Area were negatively associated with detectable dicofol levels. Our study revealed different bioaccumulation patterns of α, β-HCH and dicofol, probably due to their dissimilar period of use, and emphasize the need for assessing the exposure to frequently overlooked pollutants.

## 1. Introduction

Persistent organic pollutants (POPs) are a heterogeneous group of toxic chemicals that are highly resistant to biological degradation with a high bioaccumulation potential in living organisms, so it is believed that internal body burden of certain POPs increases over life span [1,2]. This continuous exposure might pose relevant risks to human health, since POP exposure is suspected to increase the risk of certain chronic and highly prevalent conditions, e.g., type 2 diabetes, cardiovascular disease, or cancer [3]. POPs include diverse chemical groups such as Polychlorinated Biphenyls (PCBs), which have been used in industrial applications, and Organochlorine Pesticides (OCPs) used in agriculture [4]. 

Hexachlorocyclohexanes (HCHs) are OCPs with several chemical forms, such as α-, β-, δ-, and γ-HCH, the latter also named lindane, which exerts the most effective insecticidal properties [5]. Despite these four isomers being present in the technical HCH, α-HCH is the most prevalent [6]. However, β-HCH is more resistant to degradation, more prone to bioaccumulate in living organisms and consequently is the most abundant in human tissues [7]. In fact, its half-life in fatty tissues has been estimated in the range of 7–10 years [8]. Dicofol is another OCP which has been used as an acaricide in field crops [9]. Dicofol is synthesized from technical dichlorodiphenyltrichloroethane (DDT), sharing similar chemical structures [10], and containing DDT as an impurity [11]. 

Long-term human exposure to OCPs has been documented to be related to adverse effects on health, such as neurological, reproductive, and immunological disorders [12]. Moreover, it has been associated with an increased risk of several types of cancers, such as breast, prostate, lung, stomach, colorectal, and bladder [13,14,15,16], and even with type 2 diabetes [17]. Thus, the Spanish Government banned the use of HCH containing less than 99 percent of γ-isomer in 1994 [18] and dicofol in 2009, although the latter remained in use until 2010 [19]. Moreover, three HCH isomers (α-, β-, and γ) and dicofol were included in the list of POPs of concern at the Stockholm Convention in 2009 and 2013, respectively [20,21]. Nevertheless, despite the legal restrictions on their use and production, a number of POPs can be detected in virtually all humans [22,23]. 

Regardless of their potential relevance for human health, the abovementioned OCPs have remained relatively understudied in human biomonitoring (HBM) studies in comparison to other POPs such as DDT or hexachlorobenzene (HCB), probably because of lower detection limit in human samples. 

HBM on α-, β-HCH, and dicofol has mainly focused on lipid-rich biological matrices, such as breast milk and the lipid fractions of serum and blood. Nevertheless, the concentrations in these matrices can be highly variable and not always reflect the total body burden [24]. In this regard, adipose tissue has been suggested as the most adequate matrix for estimating the long-term exposure to these chemicals [25]. However, HBM studies using adipose tissue are still relatively scarce, mostly because of the difficulties in accessibility [26,27,28,29,30,31,32]. 

In this context, this study represents a continuation of the previous work carried out by our research group, focused on the characterization of OCPs and other environmental pollutant exposures in humans, as well as their potential health outcomes [16,17,33,34,35,36]. The present study aimed to identify bioaccumulation patterns of α-, β-HCH, and dicofol in relation to sociodemographic, dietary, and lifestyle factors, in an adult cohort from Southern Spain.

## 2. Materials and Methods

### 2.1. Study Design, Characteristics of Participants, and Setting

The present cross-sectional research is framed within a larger cohort study focused on characterizing the influence of environmental factors on human health in the GraMo cohort (Granada province, Southern Spain). From July 2003 to June 2004, patients undergoing non-cancer-related surgery (41% hernias, 21% gallbladder diseases, 12% varicose veins, and 26% other conditions) in two public hospitals of Granada province (San Cecilio University Hospital in Granada city and Santa Ana Hospital in Motril) were invited to participate in the study. Both hospitals serve the population from the Center-Western and Southern areas of Granada province. The participants had to meet the following inclusion criteria: (1) age over 16 years, (2) absence of cancer diagnosis, (3) not receiving hormonal therapy, and (4) residence for the last 10 years prior to recruitment in the reference area of any of the two participant hospitals. Out of the 409 individuals contacted, a total of 387 (94.6%) participants accepted to participate in our study. Adipose tissue samples were collected from pelvic waist (50%), front abdominal wall (40%), and limbs (10%). A wider description of the GraMo cohort methodology, including characterization of other exposures, has been reported elsewhere [4,22,37,38].

All participants were informed about the study characteristics and signed a written informed consent before their inclusion in the study.

### 2.2. Sampling and α-, β-HCH, and Dicofol Analysis

Bioaccumulation of α-, β-HCH, and dicofol was estimated by analyzing their residues in adipose tissue samples collected at recruitment. 5–10 g of adipose tissue were obtained during routine surgery in the two participating hospitals. Samples were coded and stored immediately at −80 °C. Chemical analyses were performed by high-resolution gas chromatography with a mass spectrometry detector in tandem mode with a system Saturn 2000 ion trap (Varian, Walnut Creek, CA, USA) at Laboratorio Analítico Bioclínico (LAB) in Almería (Spain). The methodological aspects have been detailed elsewhere [39,40]. Lipid content in adipose tissue samples was quantified by gravimetry, as reported by Rivas et al. [40]. Normalized POP levels were expressed in nanograms per gram of lipid (ng/g lipid).

### 2.3. Data sources and Independent Variables

**Figure 1:**
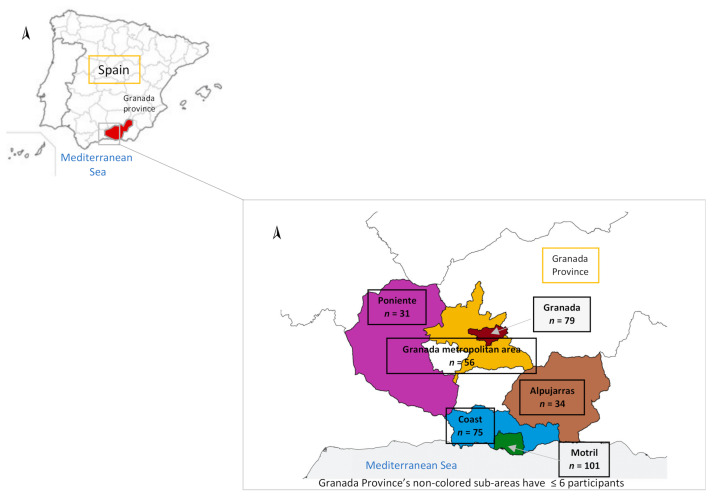
Map of Granada province (Southern Spain) with participants’ distribution by sub-areas.

During the hospital stay, a structured questionnaire was administered face-to-face to each participant by trained interviewers to gather information on socio-demographic characteristics, lifestyles, diet, and health status. The questionnaire was validated in previous studies [41,42]. Body mass index (BMI) was calculated from height and weight and expressed as Kg/m^2^. A participant was considered a smoker at recruitment at ≥1 cig/day. The residence of a participant was classified in 6 sub-areas, according to their residence: Coast region, Motril, Granada city, Granada Metropolitan Area, Poniente, and Alpujarras. The sub-areas (Alhama, Baza, and Valle de Lecrín) with ≤6 participants were excluded from the analysis. Later, the residence was recategorized in the following 3 areas: (1) Motril city and coast region and both areas in the Mediterranean coast, with large agriculture zones and greenhouses where the use of pesticides is higher; (2) Granada city and Granada Metropolitan Area, as both are urban areas with a high amount of traffic and whose main economic activity is based on industry and tourism; (3) Poniente and Alpujarras, both rural areas with less population and an agricultural tradition [4] (Figure 1). Thus, the total pesticide use in Granada province over recruitment (years 2003 and 2004) remained virtually constant (1953.03 and 2053.73 tons, respectively) [43].

The dietary information on drinking water and consumption of meat, cold meats, fats, fish, eggs, milk, cheese, vegetables, legumes, fruit, bread, and pasta was obtained from a food frequency questionnaire. Water consumption referred to the quantity consumed regardless the type of water (bottled or not, or both). A participant was considered a consumer of a specific food item at a weekly consumption of ≥1 portion.

### 2.4. Statistical Analyses

OCP concentrations in adipose tissue were described as medians, and 25th and 75th percentiles, as well as percentages over the limit of detection (LOD).

Concentrations of β-HCH were natural-log transformed to reduce the skewed distribution, and their bioaccumulation pattern in relation to sociodemographic, dietary, and lifestyle factors were identified by means of multivariable linear regression, using a combination of backward and forward variable selection techniques. In this regard, beta coefficients indicate the average change in log-transformed pollutant concentrations associated to a 1-unit increase in the continuous predictor, or the average change in log-transformed concentrations in a given level of a categorical predictor compared to the reference level. Concentrations below the LOD (15.8%) were assigned a random value between 0 and LOD. 

α-HCH and dicofol levels (% >LOD = 21.7 and 19.6%, respectively) were dichotomized (>LOD/<LOD) due to their relatively low number of samples with concentrations >LOD, and associated factors were explored by using unconditional binary logistic regression. Thus, the strength of the associations in these models are displayed as Odds Ratio (OR), i.e., odds of having detectable concentrations in a particular level of the predictor, compared to the odds of having detectable concentrations in the reference category of the predictor. In the case of continuous predictors, the OR could be interpreted as the change in the likelihood for having detectable dicofol levels per 1-unit increase in the predictor. Age, BMI, and sex (the latter in the non-stratified models) were always included in all models, regardless of their statistical significance, based on reported evidence of their association with OCP exposure. Independent variables were retained in the models based on their statistical significance, changes in R^2^ (linear regression) or pseudo-R^2^ (logistic regression), internal validity and coherence, and magnitude of effect estimates in order to explore the predictors of α-, β-HCH, and dicofol. In order to assess consistency between linear and logistic regression models, an additional sensitivity logistic regression analysis was performed using dichotomized β-HCH levels as the dependent variable to resemble the categorization for α-HCH and dicofol (80% vs. <20% of the sample). We also performed stratified analyses by sex, since several studies have evidenced differential OCP exposure levels and predictors in males and females in GraMo and other studies [22,30,38,44,45,46,47,48,49]. All statistical test were performed two-sided, and the level of statistical significance was set at *p* = 0.05. Stata 15 (Stata Corp., College Station, TX, USA, 2017) was used to perform all data analyses.

## 3. Results

### 3.1. Characteristics of the Study Population and Adipose Tissue OCP Concentrations

Characteristics of all study participants are summarized in Table 1. An additional description stratified by sex is provided as Supplementary Material (Table S1). From the 387 participants, there was a slightly higher number of males (50.9%). Mean age and BMI of the study population were 50.7 ± 17.1 years and 27.4 ± 5.4 kg/m^2^, respectively. The majority of the population (45.8%) lived in Motril city and Coast region. 

Concentrations of β-HCH, α-HCH, and dicofol were detected in the adipose tissue of 84.2%, 21.7%, and 19.6% of the participants, respectively. Β-HCH showed the highest dispersion in their levels (25th: 3.7 and 75th: 21.4 ng/g lipid), whereas α-HCH and dicofol presented the lowest dispersion (25th: <LOD and 75th: <LOD) (Table 1). Stratified by sex, females showed higher β-HCH median levels (50th: 16.1 ng/g lipid) compared to males (50th: 7.3 ng/g lipid) (Supplementary Material, Table S1).

### 3.2. Predictors of Adipose Tissue α, β-HCH, and Dicofol Concentrations

The multivariable models exploring the predictors of β-HCH, α-HCH, and dicofol exposure explained, respectively, 42%, 41%, and 15% of the variability of adipose tissue levels (Table 2). After sex-stratification, the models explained, respectively, 45%, 39%, and 26% of the variability of females’ and 41%, 47%, and 14% of males’ concentrations (Supplementary Material, Tables S2 and S3).

Age, BMI, residence in Poniente and Alpujarras, mother’s occupation in agriculture during pregnancy, and water, white fish, and milk consumption were associated with increased β-HCH concentrations. On the other hand, lower β-HCH concentrations were related to males, residence close to an agriculture area, having worked for ≥10 years in agriculture, and beer consumption. Similar associations were found in the logistic regression models using dichotomized β-HCH levels as the dependent variable (Supplementary Material, Table S4). In sex-stratified analyses, age and BMI remained as significant predictors in both males and females. Among males, increased β-HCH levels were related to residence in Poniente and Alpujarras, mother’s occupation in agriculture during pregnancy, as well as white fish and water consumption. In addition, lower β-HCH levels were associated with beer consumption, residence near an agriculture area, and occupation for ≥ 10 years in agriculture. Among females, β-HCH levels were positively related to milk and cheese consumption, and negatively with residence close to agriculture areas. 

Results from the multivariable logistic models for adipose tissue α-HCH levels are displayed in Table 2. Age, BMI, milk consumption, and mother’s occupation in agriculture during pregnancy were associated with increased odds of having detectable α-HCH concentrations, whereas males and residents in Granada city and Granada Metropolitan Area, Poniente, and Alpujarras showed lower odds (Table 2). After sex-stratification, detectable α-HCH levels were positively associated with age in both sexes. In addition, BMI (in females), mother’s occupation in agriculture during pregnancy (in males), as well as meat consumption ≥2 portions/week (in males) were positively associated with increased odds of detectable α-HCH concentrations. Furthermore, residence in Granada city and Granada Metropolitan Area, Poniente, and Alpujarras showed a negative association with detectable α-HCH concentrations both in males and females. Living close to an agriculture area (in females) was negatively associated with α-HCH levels (Supplementary Material, Tables S2 and S3). 

Finally, lower odds of detectable dicofol levels were found in smokers, white fish and water consumers, residents in Granada, Granada Metropolitan area, Poniente, Alpujarras, and those living close to an agriculture area (Table 2). In the sex-stratified analyses, white fish intake, and residence close to an agriculture area remained negatively associated with detectable dicofol levels in females, whereas negative associations were found for water intake and smoking in males. The previously observed negative association with residence in Granada city and Granada Metropolitan Area remained significant in both sexes (Supplementary Material, Tables S2 and S3). 

In order to assess the potential confounding effect of adipose tissue origin on the results, sensitivity analyses were performed by adjusting every model by adipose tissue location (pelvic waist, front abdominal wall, limbs), with no relevant change in the observed associations (data not shown).

## 4. Discussion

In this study we observed markedly different bioaccumulation patterns in adipose tissue of the selected HCH isomers and dicofol, both in their magnitude and in the determinants of their concentrations. A cluster of factors might explain these findings, including dissimilar uses, chemical properties, and the different time frames when the use of these chemicals was permitted in the region. The present research was prompted by our previous findings on differential predictors of other OCPs concentrations in this cohort, as well as their potential long-term health effects that might be produced as a consequence of the exposure to individual chemicals, but also to mixtures of them [17,22,36,38,50]. Therefore, careful analysis of individual chemical concentrations, even those at low detection rates, is warranted as a first step for exposure assessment. 

A summary of HBM studies on α-HCH, β-HCH, and dicofol concentrations published during the last 10 years is shown in Supplementary Material, Table S5. There are still a few HBM studies using adipose tissue [26,27,28,29,30,31,32], breastmilk [51,52,53,54,55,56,57], placenta [58], and meconium [59] as biological matrices to assess the OCPs exposure compared to the number of studies using serum [45,46,47,48,60,61,62,63,64,65,66,67,68,69,70,71,72,73,74,75,76,77,78,79,80,81,82,83,84,85,86,87,88,89,90,91,92,93,94,95,96,97], blood [98,99,100,101,102,103,104,105,106], and plasma [5,49,107,108,109,110,111,112,113,114]. Moreover, two studies used two biological matrices such as serum and placenta [115] or serum and omentum fat [116]. In general, in comparison to those countries where α, β-HCH were no longer used at the recruitment moment [29,31,32,48], we found lower adipose tissue α, β-HCH concentrations in the GraMo cohort, although we also observed a β-HCH higher concentration in other studies [32,116]. Regarding dicofol, the levels in our population were higher than those found in a Chinese population, despite the fact that in both countries dicofol was not yet prohibited at the time of recruitment of participants [27]. Thus, the discrepancies found in levels could be explained by differences on lifestyles, diet, and occupational/environmental exposure among different populations, as described in previous research [117,118]. 

In general, concentrations of β-HCH were detected in a higher number of participants than dicofol and α-HCH, which could be due to the higher persistence in the environment compared to dicofol [119,120,121]. Indeed, the estimated amount of technical HCH used in Spain from 1948 to 1997 was between 100,000 to 1,000,000 million tones [122], whereas the dicofol usage in the year 2000 was 125,000 kg/year [123]. Among HCH isomers, adipose tissue β-HCH levels were found at a substantially higher detection rate than α-HCH, probably because it exerts a markedly high resistance to chemical degradation both in the environment as living organisms [124], as found in previous research [27,29,30,32]. In the same way, this was observed in several studies that used other biological matrices different to adipose tissue such as blood [99,100,101,102,103,106], serum [45,72,76,77,81,86,87,125], plasma [49,58,109], and breast milk [52,53,55,57]. 

In our study population, females had higher α- and β-HCH levels compared to males, which is in agreement with the results of multivariable models and some HBM studies using adipose tissue in Danish and Japanese populations [30,44]. These gender-related differences could be related to both social and physiological aspects. Indeed, cytochrome CYP450 (family of enzymes involved in OCP metabolism) activity has been reported to be lower in females than males [126,127]. Moreover, α- and β-HCH concentrations could be influenced by the amount of body fat [128]. These findings might also be related to specific gender-related roles and behaviors in the population [22]. For example, females working in agriculture activities traditionally have lower salaries, status, and receive less education on safety measures in comparison to males [129], thus being at a higher risk of pesticide exposure [130]. Despite maternal milk and giving birth being acknowledged as major excretion routes for POPs [5,47,131,132], these variables were not associated with adipose tissue concentrations in our population (data not shown). Nevertheless, our sample size was not large enough to stratify the analyses by breastfeeding or number of children. 

Our observed differential associations of age with α- and β-HCH levels (positive) and dicofol (null) highlight the relevance of early exposures to OCPs. Interestingly, HCHs had been used since the 1940s [133], but were already banned at recruitment. On the contrary, dicofol production started in the 1990s [134] and was already in use in Spain at recruitment. Thus, in the case of HCHs, the results might relate to a “cohort effect”, by which older people born prior to prohibition of production and use of POPs could have a higher body burden of these chemicals compared to younger individuals [135], which is not the case for dicofol. 

In line with the abovementioned, BMI was positively associated with adipose tissue α-, β-HCH concentrations, but not with dicofol. These results highlight the relevance of dietary exposure to persistent chemicals, particularly to those already banned, since individuals with increased BMI are likely to consume more fatty foods [6,28], which are in turn considered the main source of exposure in non-occupational settings [28,66]. This idea is supported by positive associations in our population of α and/or β-HCH levels with the consumption of fatty foods from animal origin such as milk, cheese, and fish. In fact, dicofol concentrations were not positively associated with dietary habits. Moreover, water consumption was positively related to the most persistent HCH congener (β-HCH), probably because high food ingestion is frequently associated with increased fluid consumption [136], such as water. The negative association of α-HCH with water consumption might be either a chance finding or influenced by third variables not accounted for in this study, since levels of this congener might also be related to recent occupational exposure.

Our observed negative and null associations of β-HCH and dicofol levels with occupational activities might be related to the banning of HCH in 1994 in Spain [18] 10 years prior recruitment time, so that agricultural activities were no longer a relevant exposure source. Indeed, this finding was confirmed with the lower β-HCH and dicofol levels in those who lived close to agriculture sites compared to those that did not. Lastly, we did not observe any clear association of OCP exposure with residence, probably due to the high heterogeneity in the participants’ residence. Therefore, more research on this last issue is needed, including larger populations. 

The use of adipose tissue as an exposure assessment matrix represents a strength of the present study, since adipose tissue is likely to present lower point variability in comparison to other biological matrices [25]. Despite the relatively limited sample size, the majority of the associations in multivariable models seemed robust and plausible. Since the present research was conceived as an exploratory research in a specific population sample, further studies are warranted to confirm if our findings apply to other populations. Characterization of levels and sources of exposure is crucial in the ongoing process of exposure assessment in the GraMo cohort, and even more important considering previously reported associations of OCP with relevant health outcomes in GraMo [16,17,33,34,35,36]. This can be particularly relevant for chemicals such as dicofol and α-HCH, which are frequently overlooked in exposure-disease modeling based on their lower detection rates, but that might contribute to an overall mixture effect [137]. 

## 5. Conclusions

Our study revealed different bioaccumulation patterns of α, β-HCH, and dicofol in the analyzed population, probably related to their dissimilar periods of use. This should be considered in the evaluation of health implications of chemicals mixtures, since the specific relationship with potential confounders must be carefully considered in the models. In addition, our results emphasize the need for assessing the exposure to frequently overlooked pollutants, as well as the relevance of adipose tissue as a matrix for exposure assessment. 

## Figures and Tables

**Table 1:** Characteristics of the study population (*n* = 387).

Variable			
Sex, *n* (%)			
Females		190 (49.1)	
Males		197 (50.9)	
Age (years), mean ± SD		50.7 ± 17.1	
Body mass index (Kg/m^2^), mean ± SD		27.4 ± 5.4	
Normal weight (<25 kg/m^2^), *n* (%)		137 (35.4)	
Overweight (25-≤30 kg/m^2^), *n* (%)		167 (43.2)	
Obesity (>30 kg/m^2^), *n* (%)		83 (21.4)	
Residence, *n* (%)			
Coast, Motril		176 (45.5)	
Granada city, Granada Metropolitan Area		135 (34.9)	
Poniente, Alpujarras		65 (16.8)	
Others		8 (2.1)	
Missing		3 (0.8)	
Residence near to agricultural area ≤2000 m (yes), *n* (%)		216 (55.8)	
Residence near to greenhouse ≤2000 m (yes), *n* (%)		72 (18.6)	
Occupation in agriculture (≥10 years) (yes), *n* (%)		145 (37.5)	
Occupation in industry (≥10 years) (yes), *n* (%)		55 (14.2)	
Mother’s occupation during pregnancy, *n* (%)			
Housewife		279 (72.1)	
Agricultural worker		51 (13.2)	
Others		57 (14.7)	
Current smoker (yes), *n* (%)		126 (32.6)	
White fish consumption (yes), *n* (%)		304 (79.2)	
Meat consumption			
≤2 portions/week		140 (36.2)	
>2 portions/week		243 (62.8)	
Missing		4 (1.0)	
Milk consumer (yes), *n* (%) a		339 (90.4)	
Cheese consumer (yes), *n* (%) a		359 (93.3)	
Vegetable consumption, *n* (%)			
<2 portions/week		105 (27.1)	
≥2 portions/week		278 (71.8)	
Missing		4 (1.0)	
Beer consumption (glasses/week), mean ± SD		3.1 ± 7.9	
Water consumption (glasses/day), mean ± SD		5.6 ± 3.9	
Variable, ng/g lipid	25th	50th	75th
β-HCH	3.7	10.6	21.4
α-HCH	<LOD	<LOD	<LOD
Dicofol	<LOD	<LOD	<LOD
Variable		*n* (%)	
β-HCH (>LOD)		326 (84.2)	
α-HCH (>LOD)		84 (21.7)	
Dicofol (>LOD)		76 (19.6)	

β-HCH: β-Hexachlorocyclohexane; α-HCH: α-Hexachlorocyclohexane; LOD: limit of detection; SD: standard deviation. ^a^: Consumer is referred to intake of any amount of milk or cheese per week.

**Table 2:** Predictors of adipose tissue β-HCH, α-HCH, and dicofol concentrations in GraMo cohort (*n* = 387).

	β-HCH ^a^ (R^2^ = 0.42)		α-HCH ^b^ (pseudo-R^2^ = 0.41)		Dicofol ^b^ (pseudo-R^2^ = 0.15)	
	β (95% CI)	*p*-Value	OR (95% CI)	*p*-Value	OR (95% CI)	*p*-Value
Age (years)	0.06 (0.05, 0.07)	<0.001	1.10 (1.06, 1.12)	<0.001	0.99 (0.97, 1.00)	0.185
Sex						
Females	1.00 (ref.)		1.00 (ref.)		1.00 (ref.)	
Males	−0.67 (−1.02, −0.33)	<0.001	0.27 (0.12, 0.59)	0.001	1.18 (0.64, 2.17)	0.594
Body mass index (Kg/m^2^)	0.07 (0.04, 0.10)	<0.001	1.10 (1.02, 1.17)	0.013	1.01 (0.96, 1.07)	0.649
Residence						
Coast, Motril city	1.00 (ref.)		1.00 (ref.)		1.00 (ref.)	
Granada city, Granada Metropolitan Area	−0.12 (−0.50, 0.26)	0.540	0.03 (0.01, 0.09)	<0.001	0.18 (0.09, 0.39)	<0.001
Poniente, Alpujarras	0.59 (0.12, 1.06)	0.013	0.16 (0.06, 0.41)	<0.001	0.31 (0.12, 0.76)	0.011
Residence near to agricultural area ≤2000 m						
No	1.00 (ref.)		1.00 (ref.)		1.00 (ref.)	
Yes	−0.64 (−0.98, −0.30)	<0.001	0.52 (0.24, 1.13)	0.098	0.52 (0.28, 0.96)	0.035
Residence near to greenhouse area ≤2000 m						
No					1.00 (ref.)	
Yes					0.50 (0.23, 1.10)	0.083
Mother’s occupation during pregnancy						
Housewife	1.00 (ref.)		1.00 (ref.)		1.00 (ref.)	
Agricultural worker	0.76 (0.26, 1.27)	0.003	3.27 (1.35, 7.94)	0.009	0.53 (0.21, 1.32)	0.171
Others	0.21 (−0.26, 0.67)	0.384	1.07 (0.33, 3.50)	0.907	1.02 (0.43, 2.38)	0.969
Occupation in agriculture ≥ 10 years						
No	1.00 (ref.)				1.00 (ref.)	
Yes	−0.49 (−0.87, −0.10)	0.013			1.54 (0.79, 2.99)	0.207
Occupation in industry ≥ 10 years						
No	1.00 (ref.)					
Yes	0.12 (−0.34, 0.59)	0.605				
Water consumption (glasses/day)	0.05 (0.01, 0.10)	0.020	1.08 (0.98, 1.18)	0.124	0.91 (0.83, 0.99)	0.024
Beer consumption (glasses/week)	−0.02 (−0.04, 0.00)	0.037	0.95 (0.88, 1.03)	0.238		
Current smoker						
No					1.00 (ref.)	
Yes					0.49 (0.25, 0.99)	0.048
White fish consumption						
No	1.00 (ref.)				1.00 (ref.)	
Yes	0.47 (0.06, 0.87)	0.025			0.38 (0.19, 0.74)	0.004
Meat consumption						
<2 portions/week	1.00 (ref.)		1.00 (ref.)		1.00 (ref.)	
≥2 portions/week	0.17 (−0.16, 0.50)	0.313	1.48 (0.72, 3.06)	0.288	0.69 (0.38, 1.28)	0.239
Milk consumer ^c^						
No	1.00 (ref.)		1.00 (ref.)			
Yes	0.70 (0.14, 1.27)	0.014	3.50 (1.03, 11.88)	0.045		
Cheese consumer ^c^						
No	1.00 (ref.)					
Yes	0.56 (−0.07, 1.19)	0.081				
Vegetables consumption						
<2 portions/week	1.00 (ref.)		1.00 (ref.)			
≥2 portions/week	−0.28 (−0.64, 0.08)	0.121	0.67 (0.31, 1.48)	0.324		

^a^: Multivariable linear regression analysis. Dependent variable: log-transformed concentrations (ng/g lipid). ^b^: Multivariable logistic regression analysis. Dependent variable: dichotomized concentrations (>limit of detection vs. <limit of detection). ^c^: Consumer is referred to intake of any amount of milk or cheese per week. β-HCH: beta-hexachlorocyclohexane; α-HCH: alpha-hexachlorocyclohexane. β: Beta coefficient; CI: Confidence interval; OR: Odds Ratio; Ref.: reference category.

## Data Availability

The data presented in this study are available on request from the corresponding author.

## Supplementary Materials

The following supporting information can be downloaded at: https://www.mdpi.com/article/10.3390/ijerph19063344/s1, Table S1: Characteristics of the study population according to sex (*n* = 387); Table S2: Predictors of adipose tissue β-HCH, α-HCH and dicofol concentrations among females from GraMo cohort (*n* = 190); Table S3: Predictors of adipose tissue β-HCH, α-HCH and dicofol concentrations among males from GraMo cohort (*n* = 197); Table S4: Predictors of adipose tissue β-HCH concentrations in GraMo cohort according to the logistic multivariable model; Table S5: Review of studies on human biomonitoring of β-HCH, α-HCH and dicofol levels in the last 10 years.
