# Supplementary material for: Differential Bioaccumulation Patterns of α, β-Hexachlorobenzene and Dicofol in Adipose Tissue from the GraMo Cohort (Southern Spain)
Source: Int J Environ Res Public Health. 2022 Mar 11;19(6):3344. doi: 10.3390/ijerph19063344 (PMC8954870; doi:10.3390/ijerph19063344)
Supplement: Supplementary file 1 [file ijerph-19-03344-s001.zip › ijerph-1601083-supplementary.pdf]

International Journal of
Environmental Research and Public Health

MDPI

Supplementary Material

Table S1. Characteristics of the study population according to sex (*n* = 387).

Variable		Females (*n* = 190)			Males (*n* = 197)	
**Age (years), mean ± SD**		50.6 ± 16.7			50.8 ± 17.5	
**Body mass index (Kg/m²), mean ± SD**		27.4 ± 2.9			27.4 ± 4.9	
Normal weight (<25 kg/m^2), *n* (%)		78 (41.0)			59 (29.9)	
Overweight (25-≤30 kg/m^2), *n* (%)		67 (35.3)			100 (50.8)	
Obesity (>30 kg/m^2), *n* (%)		45 (23.7)			38 (19.3)	
**Residence, *n* (%)**						
Coast, Motril City		101 (53.2)			75 (38.1)	
Granada City, Granada Metropolitan Area		52 (27.4)			83 (42.1)	
Poniente, Alpujarras		32 (16.8)			33 (16.8)	
Others		4 (2.1)			4 (2.0)	
Missing		1 (0.5)			2 (1.0)	
**Residence near to greenhouse ≤2000 meters (yes), *n* (%)**		39 (20.5)			33 (16.8)	
**Residence near to agricultural area ≤2000 meters (yes), *n* (%)**		115 (60.5)			101 (51.3)	
**Occupation in agriculture (≥10 years) (yes), *n* (%)**		62 (32.6)			83 (42.1)	
**Occupation in industry (≥10 years) (yes), *n* (%)**		14 (7.4)			41 (20.8)	
**Mother's occupation during pregnancy, *n* (%)**						
Housewife		138 (72.6)			141 (71.6)	
Agricultural worker		27 (14.2)			24 (12.2)	
Others		25 (13.2)			32 (16.2)	
**Current smoker (yes), *n* (%)**		41 (21.6)			85 (43.2)	
**White fish consumption (yes), *n* (%)**		164 (86.8)			140 (71.8)	
**Meat consumption, *n* (%)**						
≤2 portions/week		72 (38.1)			68 (35.1)	
>2 portions/week		117 (61.9)			126 (65.0)	
**Milk consumer (yes), *n* (%)** [a]		172 (91.0)			167 (89.8)	
**Cheese consumer (yes), *n* (%)** [a]		175 (92.3)			184 (93.9)	
**Vegetable consumption, *n* (%)**						
<2 portions/week		39 (20.7)			66 (33.9)	
≥2 portions/week		149 (79.3)			129 (66.2)	
**Beer consumption (glasses/week), mean ± SD**		0.8 ± 2.0			5.3 ± 10.5	
**Water consumption (glasses/day), mean ± SD**		5.2 ± 3.0			6.0 ± 4.6	
**Variable, ng/g lipid**	**25th**	**50th**	**75th**	**25th**	**50th**	**75th**
β-HCH	2.3	16.1	30.5	6.5	7.3	14.5
α-HCH	<LOD	<LOD	<LOD	<LOD	<LOD	<LOD
Dicofol	<LOD	<LOD	<LOD	<LOD	<LOD	<LOD
**Variable**		***n* (%)**			***n* (%)**	
β-HCH (>LOD)		166 (87.4)			160 (81.2)	
α-HCH (>LOD)		58 (30.5)			26 (13.2)	
Dicofol (>LOD)		37 (19.5)			39 (19.8)	

β-HCH: β-Hexachlorocyclohexane; α-HCH: α- Hexachlorocyclohexane; LOD: limit of detection; SD: standard deviation. [a]: Consumer is referred to intake of any amount of milk or cheese per week.

Table S2. Predictors of adipose tissue β-HCH, α-HCH and dicofol concentrations among females from GraMo cohort (n = 190).

	β-HCH [a] (R^2 = 0.45)		α-HCH [b] (pseudo-R^2 = 0.39)		Dicofol [b] (pseudo-R^2 = 0.26)	
	**β (95%CI)**	***p*-value**	**OR (95%CI)**	***p*-value**	**OR (95%CI)**	***p*-value**
**Age (years)**	0.07 (0.05, 0.08)	<0.001	1.09 (1.06, 1.13)	<0.001	0.98 (0.96, 1.01)	0.213
**Body mass index (Kg/m²)**	0.05 (0.01, 0.08)	0.016	1.11 (1.03, 1.21)	0.009	1.03 (0.96, 1.11)	0.396
**Residence**						
Coast, Motril city	1.00 (ref.)		1.00 (ref.)		1.00 (ref.)	
Granada city, Granada Metropolitan Area	−0.29 (−0.79, 0.22)	0.269	0.02 (0.00, 0.09)	< 0.001	0.09 (0.02, 0.34)	<0.001
Poniente, Alpujarras	0.43 (−0.19, 1.05)	0.176	0.14 (0.04, 0.43)	0.001	0.09 (0.01, 0.78)	0.029
**Residence near to agricultural area ≤2000 meters**						
No	1.00 (ref.)		1.00 (ref.)		1.00 (ref.)	
Yes	−0.69 (−1.15, −0.22)	0.004	0.33 (0.12, 0.88)	0.027	0.27 (0.11, 0.66)	0.004
**Residence near to greenhouse area ≤2000 meters**						
No					1.00 (ref.)	
Yes					0.53 (0.18, 1.58)	0.257
**Occupation in industry ≥10 years**						
No	1.00 (ref.)					
Yes	0.63 (−0.18, 1.45)	0.123				
**White fish consumption**						
No					1.00 (ref.)	
Yes					0.21 (0.07, 0.63)	0.006
**Milk consumer [c]**						
No	1.00 (ref.)		1.00 (ref.)			
Yes	0.90 (0.14, 1.66)	0.020	2.13 (0.48, 9.44)	0.320		
**Cheese consumer [c]**						
No	1.00 (ref.)					
Yes	1.31 (0.48, 2.15)	0.002				
**Vegetable consumption**						
<2 times per week	1.00 (ref.)					
≥2 portions/week	−0.42 (−0.96, 0.12)	0.123				

[a]: Multivariable linear regression analysis. Dependent variable: log-transformed concentrations (ng/g lipid). [b]: Multivariable logistic regression analysis. Dependent variable: dichotomized concentrations (> limit of detection vs. < limit of detection). [c]: Consumer is referred to intake of any amount of milk or cheese per week. β-HCH: beta-hexachlorocyclohexane; α-HCH: alpha-hexachlorocyclohexane. β: Beta coefficient; CI: Confidence interval; OR: Odds Ratio; Ref.: reference category.

Table S3. Predictors of adipose tissue β-HCH, α-HCH and dicofol concentrations among males from GraMo cohort (*n* = 197).

	β-HCH [a] (R^2 = 0.41)		α-HCH [b] (pseudo-R^2 = 0.47)		Dicofol [b] (pseudo-R^2 = 0.14)	
	**β (95%CI)**	***p*-value**	**OR (95%CI)**	***p*-value**	**OR (95%CI)**	***p*-value**
**Age (years)**	0.05 (0.04, 0.06)	<0.001	1.12 (1.06, 1.20)	<0.001	0.99 (0.96, 1.01)	0.430
**Body mass index (Kg/m²)**	0.09 (0.04, 0.13)	<0.001	1.02 (0.91, 1.14)	0.803	1.03 (0.94, 1.13)	0.559
**Residence**						
Coast, Motril city	1.00 (ref.)		1.00 (ref.)		1.00 (ref.)	
Granada city, Granada Metropolitan Area	0.12 (−0.37, 0.61)	0.627	0.02 (0.00, 0.26)	0.002	0.26 (0.09, 0.72)	0.009
Poniente, Alpujarras	0.71 (0.10, 1.32)	0.023	0.17 (0.03, 0.79)	0.025	0.51 (0.16, 1.59)	0.244
**Residence near to agricultural area ≤2000 meters**						
No	1.00 (ref.)					
Yes	−0.54 (−0.98, −0.10)	0.017				
**Residence near to greenhouse area ≤2000 meters**						
No					1.00 (ref.)	
Yes					0.65 (0.19, 2.18)	0.486
**Mother's occupation during pregnancy**						
Housewife	1.00 (ref.)		1.00 (ref.)		1.00 (ref.)	
Agricultural worker	1.05 (0.38, 1.72)	0.002	15.19 (2.83, 81.60)	0.002	0.44 (0.11, 1.82)	0.257
Others	−0.25 (−0.82, 0.32)	0.397	0.15 (0.01, 2.31)	0.172	0.81 (0.23, 2.83)	0.748
**Occupation in agriculture ≥10 years**						
No	1.00 (ref.)				1.00 (ref.)	
Yes	−1.04 (−1.54, −0.54)	<0.001			1.72 (0.67, 4.40)	0.258
**Water consumption (glasses/day)**	0.09 (0.04, 0.14)	0.001	1.16 (0.99, 1.35)	0.062	0.88 (0.78, 0.99)	0.034
**Beer consumption (glasses/week)**	−0.04 (−0.06, −0.02)	<0.001	0.89 (0.77, 1.02)	0.089		
**Current smoker**						
No					1.00 (ref.)	
Yes					0.30 (0.12, 0.75)	0.011
**White fish consumption**						
No	1.00 (ref.)					
Yes	0.55 (0.10, 0.99)	0.018				
**Meat consumption**						
<2 portions/week	1.00 (ref.)		1.00 (ref.)		1.00 (ref.)	
≥2 portions/week	0.43 (−0.02, 0.88)	0.063	5.22 (1.16, 23.41)	0.031	0.44 (0.18, 1.07)	0.072
**Vegetable consumption**						
<2 portions/week			1.00 (ref.)			
≥2 portions/week			0.24 (0.05, 1.07)	0.061		

[a]: Multivariable linear regression analysis. Dependent variable: log-transformed concentrations (ng/g lipid). [b]: Multivariable logistic regression analysis. Dependent variable: dichotomized concentrations (> limit of detection vs. < limit of detection). β-HCH: betahexachlorocyclohexane; α-HCH: alpha-hexachlorocyclohexane. β: Beta coefficient; CI: Confidence interval; OR: Odds Ratio; Ref.: reference category.

Table S4. Predictors of adipose tissue β-HCH concentrations in GraMo cohort according to the logistic multivariable model.

	β-HCH (pseudo-R^2 = 0.36)	
	**OR (95% CI)**	***p*-value**
**Age (years)**	1.10 (1.07, 1.13)	<0.001
**Sex**		
Females	1.00 (ref.)	
Males	0.18 (0.08, 0.41)	<0.001
**Body mass index (Kg/m²)**	1.06 (0.98, 1.12)	0.152
**Residence**		
Coast, Motril	1.00 (ref.)	
Granada city, Granada Metropolitan Area	0.28 (0.11, 0.69)	0.006
Poniente, Alpujarras	1.49 (0.61, 3.64)	0.379
**Residence near to agricultural area ≤2000 meters**		
No	1.00 (ref.)	
Yes	0.26 (0.12, 0.58)	0.001
**Mother's occupation during pregnancy**		
Housewife	1.00 (ref.)	
Agricultural worker	2.10 (0.81, 5.49)	0.128
Others	0.25 (0.06, 0.98)	0.047
**Occupation in agriculture ≥10 years**		
No	1.00 (ref.)	
Yes	0.78 (0.35, 1.72)	0.538
**Occupation in industry ≥10 years**		
No	1.00 (ref.)	
Yes	0.89 (0.32, 2.52)	0.834
**Water consumption (glasses/day)**	1.05 (0.94, 1.18)	0.361
**Beer consumption (glasses/week)**	0.96 (0.88, 1.05)	0.410
**White fish consumption**		
No	1.00 (ref.)	
Yes	3.78 (1.21, 11.80)	0.022
**Meat consumption**		
<2 portions/week	1.00 (ref.)	
≥2 portions/week	1.04 (0.51, 2.10)	0.918
**Milk consumer** [a]		
No	1.00 (ref.)	
Yes	2.91 (0.756, 11.36)	0.123
**Cheese consumer** [a]		
No	1.00 (ref.)	
Yes	1.39 (0.37, 5.17)	0.623
**Vegetables consumption**		
<2 portions/week	1.00 (ref.)	
≥2 portions/week	0.27 (0.12, 0.61)	0.002

[a]: Consumer is referred to intake of any amount of milk or cheese per week. β-HCH: betahexachlorocyclohexane; CI: Confidence interval; OR: Odds Ratio; Ref.: reference category.

Table S5. Review of studies on human biomonitoring of β-HCH, α –HCH and dicofol levels in the last 10 years.

Reference	N	Study population	Location	Recruitment data	Matrix	Units	β-HCH	α-HCH	Dicofol
Arguin et al., 2010	61	Vegans, omnivores and obese men	USA	NA	Plasma	ng/g<br>ng/mL	Vegans: 6.515 ^{AM}<br>Omnivores: 5.720 ^{AM}<br>Standard: 0.045 ^{AM}<br>Fat-reduced: 0.051^{AM}<br>Fat-substituted: 0.088 ^{AM}	-	-
Grimalt et al., 2010	695	Child at birth and at 4 years	Spain	1997/98	Serum	ng/mL	Cord: 0.21^{AM}<br>Serum: 0.28^{AM}	-	-
Lu et al., 2010	262	Children aged 6–10 years	China	2008	Serum	ng/mL	-	0.0093^{AM}	-
Ociepa-Zawal et al., 2010	77	Breast cancer patients and control	Poland	NA	Adipose tissue	ng/g fat	Co: 18 ^{M}, Ca: 24 ^{M}	Co: 24 ^{M}, Ca: 7 ^{M}	-
Park et al., 2010	100	Metabolic syndrome cases and controls	Korea	2006	Serum	ng/g lipid	Co: 46.1^{AM}, Ca: 61.5 ^{AM}	-	-
Pathak et al., 2010	60	Primiparous women	India	NA	Blood	ng/mL	Full-term: 4.12 ^{AM}<br>Preterm: 9.14 ^{AM}	Full-term: 5.87 ^{AM}<br>Preterm: 6.65 ^{AM}	-
Porta et al., 2010	919	General population	Spain	2001/02	Serum	ng/g lipid	91.9^{M}	-	-
Sawada et al., 2010	603	Prostate cancer patients and controls	Japan	1990/95	Plasma	ng/g lipid	Co: 320 ^{M}, Ca: 310 ^{M}	-	-
Son et al., 2010	80	Type 2 diabetes cases and controls	Korea	2006	Serum	ng/g lipid	Co: 44.0 ^{AM}, Ca: 57.9 ^{AM}	-	-
Weldon et al., 2010	364	Mexican-American pregnant women	USA	1999/02	Serum	ng/g lipid	39.3 ^{M}	-	-
Cao et al., 2011	1438	Pregnant women	China	2008/09	Cord blood serum	ng/mL	0.45 ^{M}	0.05 ^{M}	-
Ibarluzea et al., 2011	1259	Pregnant women	Spain	2004/08	Serum	ng/g lipid	19.14 ^{GM}		
Wang et al., 2011	633	Residents in three cities from China	China	2008/09	Adipose tissue	ng/g	189, 424, 253 ^{AM}	1.81, 3.19, 1.79 ^{AM}	9.06 ^{AM}, 2.91^{AM}, 4.82 ^{AM}
Bräuner et al., 2012	245	General population	Denmark	1993/97	Adipose tissue	ng/g	♂: 51^{M}<br>♀: 61 ^{M}	-	-
Channa et al., 2012	241	Delivering women	South Africa	NA	Plasma	ng/mL	0.064 ^{M}	0.013 ^{M}	-
Dhananjayan et al., 2012	30	Agriculture and sheep wool workers	India	2009	Blood	ng/mL	9.55 ^{AM}	3.54 ^{AM}	-
Kanazawa et al., 2012	186	Pregnant women	Japan	2002/05	Blood	pg/g wet	150 ^{GM}	1.1^{GM}	-
Kaushik et al., 2012	79	Residents in Haryana state	India	1992/02	Serum	ng/mL	1992: 39 ^{AM}, 2002: 53 ^{AM}	1992: 201^{AM}, 2002: 8 ^{AM}	-

Table S5. Continued

Reference	N	Study population	Location	Recruitment data	Matrix	Units	β-HCH	α-HCH	Dicofol
Louis et al., 2012	939	Hospital women and healthy women	USA	2007/09	Omentum fat and serum	ng/g	Endom: 0.1991 ^{M}<br>Non-endom: 0.1200 ^{M}<br>Endom: 0.0063 ^{M}<br>Non-endom: 0.0063 ^{M}<br>Endom: 0.0066 ^{M}<br>Non-endom: 0.0063 ^{M}	-	-
Porta et al., 2012	378	General population	Spain	2002/06	Serum	ng/g lipid	2002: 128.9 ^{M}, 2006: 64.2 ^{M}	-	-
Rudge et al., 2012	155	Delivering women	Brazil	2007/08	Blood	ng/mL	0.029 ^{M}	0.001 ^{M}	-
Sharma et al., 2012	100	FGR cases and controls	India	2008	Maternal blood	ppb	Co: 3.97 ^{AM}, Ca: 9.02 ^{AM}	Co: 2.92 ^{AM}, Ca: 4.55 ^{AM}	-
Trejo-Acevedo et al., 2012	45	Healthy children aged 4–12 years	Mexico	2006	Plasma	ng/mL	ND (LOD = 0.3)	ND (LOD = 0.3)	-
Zhao et al., 2012	1307	Carcinoma cases and controls	China	2007/09	Serum	ng/mL	Co: 9.36 ^{M}, Ca: 10.23 ^{M}	Co: 1.38 ^{M}, Ca: 1.81 ^{M}	-
Bjermo et al., 2013	246	General population	Swiss	2010/11	Serum	ng/mL	0.078 ^{M}	-	-
Dewan et al., 2013	60	Mothers' SGA new-born	India	2009/10	Maternal blood	ng/mL	Co: 5.9 ^{AM,} Ca: 8.1 ^{AM}	Co: 4.9 ^{AM,} Ca: 5.2 ^{AM}	-
Freire et al., 2013	610	Men and women exposed population	Brazil	2003 04	Serum	ng/mL	♂: 6.00 ^{M}, ♀: 6.98 ^{M}	♂: 2.52 ^{M}, ♀: 2.60 ^{M}	-
Malarvannan et al., 2013	52	Obese individuals	Belgium	2010/12	Adipose tissue	ng/g	Visceral & subcutaneous fat: 18 ^{M}	Visceral fat: 1.0 ^{M}<br>Subcutaneous fat: 0.21 ^{M}	
Morales et al., 2013	2031	Pregnant women	Spain	2003/08	Serum	ng/g lipid	26.5 ^{GM}	-	-
Mrema et al., 2013	136	General population	Italy	NA	Serum	pmol/g lipid	♂ (N): 59 ^{M}, ♀ (N): 65 ^{M}<br>♂ (P): 303 ^{M}, ♀ (P): 327^{M}<br>♂ (M): 50 ^{M,} ♀ (M): 52 ^{M}	-	-
Porta et al., 2013	246	Exposed population	Italy	2006/07	Serum	ng/g	60.6 ^{GM}	-	-
Reid et al., 2013	167	Pregnant women	Australia	2009/11	Plasma	ng/mL	0.18 ^{AM}	-	-
Singh et al., 2013	145	Alzheimer's cases and controls	India	2010/11	Blood	ng/mL	Ca: 4.16 ^{AM}, Co: 0.25 ^{AM}	Ca: 0.25 ^{AM}, Co: 0.56 ^{AM}	-
Upson et al., 2013	818	Endometriosis cases and controls	USA	1996/01	Serum	ng/g	Co: 0.431 ^{M}, Ca: 0.519 ^{M}	-	-
Valera et al., 2013	315	General population	Canada	1992	Plasma	ng/mL	0.1^{GM}	-	-
Wang et al., 2013	54	Healthy women	China	2011	Plasma	ng/g lipid	♂: 346 ^{M}, ♀: 221 ^{M}	♂: 27.7 ^{M}, ♀: 17.8 ^{M}	-

Table S5. Continued

Reference	N	Study population	Location	Recruitment data	Matrix	Units	β-HCH	α-HCH	Dicofol
Watanabe et al., 2013	20	Cadavers	Japan	2003/04	Adipose tissue	ng/g lipid	♂: 770 ^{AM,} ♀: 950 ^{AM}	♂: 1.8 ^{AM}, ♀: 2.3 ^{AM}	-
Adlard et al., 2014	363	Canadian foreign-born mothers, Canadian born mothers, Mexican mothers	Canada, Mexico	2005/07	Plasma	ng/g lipid	7.7, 2.1, 8.3 ^{GM}	-	-
Azandjeme et al., 2014	118	Diabetics adults	West Africa	2011	Serum	ng/g lipid	2.9 ^{GM}	-	-
Ben Hassine et al., 2014	113	Hospitals individuals	Tunisia	2011/12	Serum	ng/g lipid	9.5 ^{M}	-	-
Braun et al., 2014	60	Pregnant women	USA	2003/06	Serum	ng/g lipid	1.9 ^{GM}	-	-
Curren et al., 2014	173	Primiparous women in 4 Canadian Artic centres & Canadian-foreign-born	Canada	2005/07	Plasma	ng/g lipid	3.7, 3.0^{GM}, ND, 2.4, 2.1, 7.7 ^{GM}	-	-
Freire et al., 2014	604	Rural men, premenopausal women, peri-/postmenopausal women	Brazil	2003/04	Serum	ng/mL	6.0, 6.3, 11.7 ^{M}	2.5, 2.8, 2.4 ^{M}	-
Guo et al., 2014	81	Mother-infant pairs	China	2010	Serum	ng/g	Mothers: 67.7 ^{GM}, Newborns: 33.9 ^{GM}	-	-
Itoh et al., 2014	399	Women from general population	Japan	2001/05	Serum	ng/g lipid	64 ^{M}	-	-
Lam et al., 2014	350	Boys aged 8-9 years	Russia	2003/05	Serum	ng/g lipid	168 ^{M}	-	-
Li et al., 2014	247	Mother-infant pairs	China	2010	Serum	ng/g lipid	Maternal: 27.33 ^{M} Neonatal: 13.33 ^{M}	<LOD ^{M}	-
Mørck et al., 2014	259	Mothers and schoolchildren	Danmark	2011	Serum	ng/g lipid	Mothers: 15 ^{AM} Children: 2 ^{AM}	-	-
Saoudi et al., 2014	386	General population	France	2006/07	Serum	ng/g lipid	27.0 ^{M}	0.74 ^{M}	-
Savitz et al., 2014	1921	Pregnant women	USA	1959/66	Serum	ng/mL	1.39 ^{M}	-	-
Steenland et al., 2014	89	Elderly subjects >65 years	Costa Rica	2012	Serum	ng/mL	0.71 ^{M}	-	-
Waliszewski et al., 2014	225	General population	Mexico	2010/11	Adipose tissue	ng/g	70, 109, 68 ^{AM}	-	-
Lu et al., 2015	142	Pregnant mothers	Shangai	2011/12	Breastmilk	ng/g lipid	12.9 ^{M}	0.41 ^{M}	-
Sharma et al., 2015	111	General population	Pakistan/ India	2014	Blood	ng/mL	84.56 ^{M}	7.57 ^{M}	-
Zubero et al., 2015	162	General population	Spain	2006/08	Serum	ng/g lipid	-	2006: 46.2 ^{GM} 2008: 36.2 ^{GM}	-

Table S5. Continued

Reference	N	Study population	Location	Recruitment data	Matrix	Units	β-HCH	α-HCH	Dicofol
Castillo-Castañeda et al., 2016	108	Lactating women	Mexico	NA	Breastmilk	ng/mL	11 ^{M}	2.4 ^{M}	-
Dimitriadou et al., 2016	87	Primipara and multipara mothers	Greece	2004/05	Breastmilk	ng/g lipid	40 ^{M}	<0.20 ^{AM}	-
Jeong et al., 2016	72	New-born infants	Korea	2012	Meconium	ng/g lipid	-	3.83 ^{M}	-
Achour et al., 2017	40	Patients undergoing surgery	Tunisia	2014	Adipose tissue	ng/g lipid	5.58 ^{M}	1.15 ^{M}	-
Bravo et al., 2017	698	Pregnant women	Argentina	2011/12	Maternal serum	ng/g lipid	Ushuaia: 6.8 ^{M}, Salta: 11 ^{M}	Ushuaia: 4.1 ^{M}, Salta: 0.51^{M}	-
Bjerregaard-Olesen et al., 2017	197	Pregnant women	Denmark	2011/13	Serum	ng/g lipid	2011: 2.8 ^{AM}; 2012: 2.0 ^{AM}; 2013: 2.0 ^{AM}	-	-
Do Nascimiento et al., 2017	542	Blood donors aged 18–65	Brazil	2015	Serum	ng/mL	0.28 ^{GM}	-	-
Mamontova et al., 2017	139	Mothers	Russia	1997/09	Breastmilk	ng/g lipid	-	Villages:<br>Balanansk: 6.8 ^{AM}<br>Kachug: 4.1 ^{AM}<br>Elantsy: 5.5 ^{AM}<br>Onguren: 3.4 ^{AM}<br>Khuzhir: 1.7 ^{AM}<br>Maloye: 3.2 ^{AM}<br>Bolshoye: 3.2 ^{AM}<br>Goloustnoe: 3.2 ^{AM}<br>Tankhoy: 1.2 ^{AM}<br>Towns:<br>Usol'e-Sibirskoe: 2.6 ^{AM}<br>Irkutsk: 3.1 ^{AM}<br>Schelekhovo: 5.6 ^{AM}<br>Ust'-Ilimsk: 2.0 ^{AM}<br>Baikal'sk: 3.1 ^{AM}<br>Bratsk: 3.9 ^{AM}<br>Petrovsk-Zabaikal'skiy: 2.3 ^{AM}	-
Ramos et al., 2017	1880	General population	Spain	2009/10	Serum	ng/g lipid	♂: 100.6 ^{GM}<br>♀: 124.5 ^{GM}	-	-
Thomas et al., 2017	12175	General population	Australia	2002/13	Serum	ng/g lipid	2002/03: 10.1 ^{AM}<br>2008/09: 7.1 ^{AM}<br>2010/11: 4.3 ^{AM}<br>2012/13: 5.0 ^{AM}	-	-

Table S5. Continued

Reference	N	Study population	Location	Recruitment data	Matrix	Units	β-HCH	α-HCH	Dicofol
Wang et al., 2017	1923	General population	China	2014	Serum	ng/g lipid	Cities: Huaihua: 139.95 ^{GM} Yitong: 100.83 ^{GM} Ganzi: 52.34 ^{GM} Lingshui:34.61 ^{GM} Weifang: 168.52 ^{GM}	Cities: Huaihua:0.38 ^{GM} Yitong:1.31 ^{GM} Ganzi:1.23 ^{GM} Lingshui:0.91 ^{GM} Weifang:1.24 ^{GM}	-
Coakley et al., 2018	734	General population aged 19–64	New Zealand	2011/12	Serum	ng/g lipid	7.0 ^{GM}	-	-
Eroglu et al., 2018	100	Agricultural workers women	Turkey	2013	Breastmilk	ng/g lipid	147.36 ^{M}	25.75 ^{M}	-
González-Alzaga et al., 2018	133	Children living in farming communities	Spain	2011	Serum	ng/g lipid	21 ^{GM}		
Harmouche-Karaki et al., 2018	314	University students	Lebanon	2013/15	Serum	ng/g lipid	8.6 ^{GM}	-	-
Kim et al., 2018	180	Residents over 40 years of age	Korea	2006	Serum	ng/g lipid	HP: 37.3 ^{M}, MS: 52.2 ^{M} T2DM: 52.3 ^{M}	-	-
Li et al., 2018	100	Blood donors	China	NA	Plasma	ng/mL	♂: 2.65 ^{M}, ♀: 2.85 ^{M}	♂: 3.64 ^{M}, ♀: 3.74 ^{M}	<LOD ^{M}
Song et al., 2018	40	Healthy lactation women	China	NA	Breastmilk	ng/g lipid	105^{AM}	-	-
Toichuev et al., 2018	508	Women who have just given birth	Kyrgyzstan	2011/15	Placenta	ng/g	77.0 ^{M}	4.6 ^{M}	-
Zong et al., 2018	793	Women with T2DM and controls	USA	1995/2000	Blood	ng/g lipid	Co: 9.84 ^{AM}, Ca: 14.3 ^{AM}	-	-
Aerts et al., 2019	206	Breastfeeding women	Belgium	2014	Breastmilk	ng/g lipid	2.40 ^{M}	-	-
Bravo et al., 2019	250	Pregnant women	Russia	2014/15	Serum	ng/g lipid	38 ^{AM}	3.3 ^{AM}	-
Luzardo et al., 2019	121	General population	Romania	2017/08	Serum	ng/mL	0.5 ^{M}	-	-
Yin et al., 2019	79	Volunteer mothers	China	2016	Maternal serum, cord serum and placenta	ng/g lipid	Maternal serum: 7.31 ^{M} Cord serum: 4.35 ^{M} Placenta: 3.21 ^{M}	Maternal serum: 1.62 ^{M} Cord serum: 0.85 ^{M} Placenta: 0.19 ^{M}	-
Narduzzi et al., 2020	602	Exposed participants	Italy	2013/15	Serum	ng/g lipid	71 ^{M}	-	-

β-HCH: β-Hexachlorocyclohexane; α-HCH: α- Hexachlorocyclohexane; NA: Non available; LOD: Limit of detection; ND: Non detected; ^{GM} Geometric Mean; ^{AM} Arithmetic mean; ^{M} Median; HP: Healthy participants; MS: Metabolic Syndrome; T2DM: Type 2 Diabetes Mellitus; ♂: men, ♀: women; Co: controls; Ca: cases; endom: endometriosis; non-endom: non-endometriosis; N: novafeltria; P: Pavia; M:Milan; V:Veracruz; P: Puebla; T: Tabasco; FGR: Fetal Growth Restriction
